# KOPS-guided DNA translocation by FtsK safeguards *Escherichia coli* chromosome segregation

**DOI:** 10.1111/j.1365-2958.2008.06586.x

**Published:** 2008-12-31

**Authors:** Viknesh Sivanathan, Jenny E Emerson, Carine Pages, François Cornet, David J Sherratt, Lidia K Arciszewska

**Affiliations:** 1 Department of Biochemistry, University of OxfordOxford OX1 3QU, UK; 2 Laboratoire de Microbiologie et de Génétique MoléculaireCNRS UMR5100, Toulouse, France

## Abstract

The septum-located DNA translocase, FtsK, acts to co-ordinate the late steps of *Escherichia coli* chromosome segregation with cell division. The FtsK γ regulatory subdomain interacts with 8 bp KOPS DNA sequences, which are oriented from the replication origin to the terminus region (*ter*) in each arm of the chromosome. This interaction directs FtsK translocation towards *ter* where the final chromosome unlinking by decatenation and chromosome dimer resolution occurs. Chromosome dimer resolution requires FtsK translocation along DNA and its interaction with the XerCD recombinase bound to the recombination site, *dif*, located within *ter*. The frequency of chromosome dimer formation is ∼15% per generation in wild-type cells. Here we characterize FtsK alleles that no longer recognize KOPS, yet are proficient for translocation and chromosome dimer resolution. Non-directed FtsK translocation leads to a small reduction in fitness in otherwise normal cell populations, as a consequence of ∼70% of chromosome dimers being resolved to monomers. More serious consequences arise when chromosome dimer formation is increased, or their resolution efficiency is impaired because of defects in chromosome organization and processing. For example, when Cre–*loxP* recombination replaces XerCD–*dif* recombination in dimer resolution, when functional MukBEF is absent, or when replication terminates away from *ter*.

## Introduction

Our understanding of bacterial chromosome segregation mechanisms remains enigmatic. In contrast to uncertainties about the processes that lead to bulk chromosome segregation in vegetative cells, double-strand DNA translocases of the FtsK/SpoIIIE family have been demonstrated to actively participate in the late stages of chromosome segregation and in transferring DNA into the *Bacillus subtilis* forespore (Wu and Errington, 1994; Sherratt *et al*., 2001; Bigot *et al*., 2007).

In *Escherichia coli*, FtsK, which localizes to the forming septum, acts as part of a recruitment pathway for complete divisome assembly (Goehring and Beckwith, 2005). The *E. coli* chromosome is segregated sequentially soon after replication, with the bulk of the chromosome segregated away from the septal region before active FtsK translocase at the closing septum is established (Kennedy *et al*., 2008). Only when chromosomal DNA is trapped at the septum does FtsK translocase facilitate completion of chromosome unlinking and segregation.

*Escherichia coli* FtsK contains three domains within its 1329 aa residues (reviewed in Bigot *et al*., 2007). The N-terminal integral membrane domain anchors FtsK to the septum and functions in cytokinesis (Draper *et al*., 1998). The C-terminal domain is a DNA translocase that can be divided into three subdomains; α and β form the motor, while γ is a regulatory domain that binds DNA and interacts with the XerCD recombinase (Barre *et al*., 2000; Massey *et al*., 2006; Sivanathan *et al*., 2006; Yates *et al*., 2006). The N- and C-terminal domains are joined by a large internal linker domain whose precise function remains unclear. DNA translocation by FtsK is directional and is guided by octameric sequences (KOPS in *E. coli* and SRS in *B. subtilis*) that show biased orientation on the leading strand of each replication arm (replichore), with their orientation switching at the *dif* locus (Bigot *et al*., 2005; Levy *et al*., 2005; Hendrickson and Lawrence, 2006; Becker and Pogliano, 2007; Ptacin *et al*., 2008). A consequence of this guided translocation is that the chromosome terminus region (*ter*) is translocated towards the closing septum. The winged helix γ subdomain recognizes KOPS (Sivanathan *et al*., 2006; Löwe *et al*., 2008). This interaction leads to the assembly of a FtsK hexamer on one side of KOPS, thereby imposing directional loading and translocation of FtsK along DNA, thus resulting in unidirectional translocation of each replichore arm.

Trapped DNA at the septum occurs predominantly when chromosome dimers, which arise through crossing over by homologous recombination, fail to be completely segregated to sister cells. Resolution of *E. coli* chromosome dimers results from a crossover introduced by the XerCD site-specific recombinase at *dif*, located in *ter* (Blakely *et al*., 1991; 1993; Kuempel *et al*., 1991). This reaction requires FtsK to directly interact with XerCD to activate dimer resolution (and to facilitate decatenation). Furthermore, FtsK translocation is necessary to bring sister *dif* sites together in a way that entraps no intervening DNA; thereby ensuring that the products of recombination do not contain catenane or knot links (Recchia *et al*., 1999; Steiner *et al*., 1999; Ip *et al*., 2003; Yates *et al*., 2006; Grainge *et al*., 2007). Therefore, the engagement of FtsK with the *E. coli* chromosome plays a crucial role in chromosome dimer resolution and the subsequent timely chromosome segregation to sister cells. Here we investigate the importance in *E. coli* chromosome processing of KOPS-guided directionality during translocation along DNA by FtsK. We show that failure to recognize KOPS during FtsK translocation has only modest consequences for fitness of wild-type cells, with ∼70% of chromosome dimers that arise as a consequence of homologous recombination being resolved to monomers. In contrast, directionality in FtsK translocation becomes important for maintaining fitness when timely chromosome processing is impaired in MukBEF^−^ and Tus^−^ cell populations, and when Cre–*loxP* recombination replaces XerCD recombination at *dif*.

## Results and discussion

### FtsK mutants defective in KOPS recognition

The 69 aa C-terminal FtsK γ subdomain is a winged helix that recognizes KOPS DNA (Sivanathan *et al*., 2006). Single aa substitutions in the DNA recognition helix of a biochemically active translocase, FtsK_50C_, have been characterized (Aussel *et al*., 2002; Sivanathan *et al*., 2006). FtsK_50C_[R1300A] and FtsK_50C_[E1303A] are defective in KOPS recognition while fully supporting XerCD–*dif* recombination in plasmid resolution assays *in vivo* or *in vitro*; reactions that require DNA translocation and activation of Xer recombination by FtsK. However, these variants retained residual KOPS recognition when assayed in triplex displacement assays (Sivanathan *et al*., 2006). In an attempt to construct a variant fully defective in KOPS recognition, we now substituted three residues that were previously shown to contribute to KOPS recognition, generating FtsK_50C_[R1300A, E1303A, E1306A], hereafter named FtsK_50C_[blind].

**Fig. 1:**
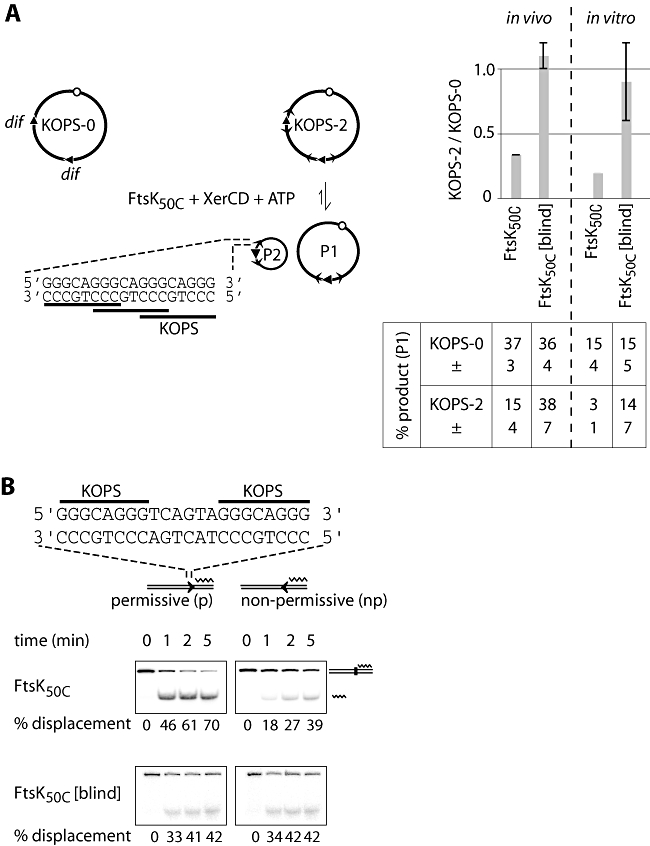
Loss of KOPS recognition. A. *dif* recombination assay. The KOPS-0 plasmid is a pSC101 derivative carrying two directly oriented *dif* sites separated by a kanamycin-resistance gene cassette. The KOPS-2 plasmid is the same as KOPS-0, other than having three overlapping KOPS in the non-permissive orientation bounding each *dif* site. XerCD-mediated *dif* recombination generates two circular molecules, P1 and P2, with only P1 being able to replicate *in vivo*. Left: schematic of the reaction. Right: histogram showing ratios of recombination levels of KOPS-2 to KOPS-0 for FtsK_50C_ and FtsK_50C_[blind]*in vivo* and *in vitro*. Values below chart show means and SD from two independent experiments. B. Triplex displacement assay. The 2.9 kb DNA substrates contain a triplex-forming sequence 15 bp from one end. The substrates contained two non-overlapping KOPS in either the permissive (p) or non-permissive (np) orientation 60 bp from the triplex forming site. Displacement of a ^32^P-labelled triplex-forming oligonucleotide carrying a four-nucleotide flap (jagged line) was monitored for 5 min.

FtsK_50C_[blind] was first tested *in vivo* for its ability to support XerCD recombination between directly repeated *dif* sites on a plasmid that either lacked KOPS (KOPS-0) or had pairs of KOPS in the non-permissive orientation bounding both *dif* sites (KOPS-2) (Fig. 1A). Preferential loading of FtsK at a given KOPS on the KOPS-2 substrate leads to translocation away from the proximal *dif* site and can result in collisions with converging FtsK hexamers loaded randomly or at other KOPS, thereby leading to a reduction in substrate resolution, when compared with the KOPS-0 plasmid (Sivanathan *et al*., 2006; Löwe *et al*., 2008). FtsK_50C_[blind] supported resolution of both plasmids with similar efficiencies, at levels of recombination similar to those observed with wild-type FtsK_50C_ on the KOPS-0 substrate, a result consistent with FtsK_50C_[blind] failing to recognize KOPS. The same results were obtained in *in vitro* resolution assays, thereby confirming that FtsK_50C_[blind] fails to recognize KOPS, yet is proficient for DNA translocation and activation of Xer recombination.

The relative abilities of FtsK_50C_[blind] and FtsK_50C_ to displace a radiolabelled oligonucleotide in triplex displacement assays were then compared (Fig. 1B). In these assays, preferential loading of an FtsK derivative at KOPS leads to translocation towards the triplex [permissive orientation (p)], or away from the triplex [non-permissive orientation (np)] upon addition of ATP; the latter event results in lowered amounts of triplex displaced. The ability of FtsK_50C_[blind] to displace the triplex oligonucleotide with equal efficiencies on both substrates, as assessed by the initial amount of displacement in the first minute, confirms that FtsK_50C_[blind] is unable to recognize KOPS; triplex displacement on these substrates must arise from random loading onto DNA.

### Effects of KOPS blindness on cell growth

To test the biological consequences of the inability of FtsK to recognize KOPS, the *ftsK[blind]* allele was introduced into the *ftsK* gene, expressed from its endogenous chromosomal position. A chromosomal derivative containing the *ftsK[E1303A]* allele, which is partly functional in KOPS recognition, was also constructed. The properties of cells containing these derivatives were compared with cells with wild-type *ftsK*, and with *ftsK[Δγ]*, which is translocation competent but is unable to recognize KOPS or activate chromosome dimer resolution (Sivanathan *et al*., 2006), and with *ftsK[ΔLC]*, which is lacking the linker and the C-terminal translocase domain (deleted for aa residues 211–1329; Bigot *et al*., 2004) and therefore is totally deficient in DNA translocation and XerCD–*dif* recombination activation. FtsK[blind] expressed from the chromosome supported XerCD–*dif* recombination *in vivo* on the KOPS-0 and KOPS-2 plasmids (not shown), and excision of a *dif*-*lacI*-*dif* cassette inserted at *dif* in the chromosome (later).

**Fig. 2:**
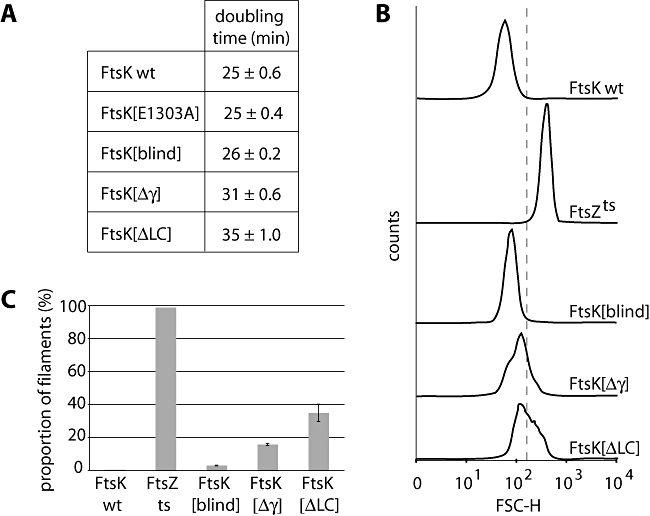
Growth of strains carrying FtsK variants. A. The doubling time during exponential growth was determined in LB at 37°C by monitoring A_600_. B. Cell length distribution of cells for each strain during exponential growth was determined by FACS using light scattered by cells in the forward (FSC-H) and side (SSC-H) directions. *ftsZ*^ts^ cells exponentially grown at 30°C were shifted to 42°C for 2 h prior to FACS analysis. C. The histogram shows the level of filaments formed by each strain as determined by FACS (mean and SD from two independent experiments).

We first determined doubling times in cultures of strains carrying the wild-type and variant *ftsK* alleles in Luria–Bertani (LB) medium. Variants that were only defective in KOPS recognition, *ftsK[E1303A]* and *ftsK[blind]*, showed no significant difference in growth rates relative to the *ftsK*^+^ strain (doubling times of 25–26 min; Fig. 2A). Growth rates of *ftsK[blind]* and wild-type strains were also not significantly different in minimal medium (not shown). The *ftsK[Δγ]* and *ftsK[ΔLC]* strains had longer doubling times in LB (31 and 35 min respectively), consistent with a minority of cell divisions producing inviable cells as a result of unresolved chromosome dimers (Steiner and Kuempel, 1998; Perals *et al*., 2000; Fig. 2A).

A failure to segregate DNA from midcell during septation, which occurs predominantly when chromosome dimers are left unresolved, leads to SOS-induced filamentation of cells (Hendricks *et al*., 2000; Prikryl *et al*., 2001). The level of filamentation in a population of cells therefore reflects the proportion of cells defective for chromosome dimer resolution. Filamentation in each FtsK variant was monitored by flow cytometry of exponentially growing cells (LB), using filamentation of a FtsZ^ts^ strain grown at 42°C as a positive control (Fig. 2B and C). The proportion of filaments in a *ftsK[Δγ]* culture was ∼16%, a value consistent with chromosomal dimers being produced once every six generations (Steiner and Kuempel, 1998; Perals *et al*., 2000). The proportion of filaments in a *ftsK[ΔLC]* was ∼34%, a result that agrees with the report that loss of the linker domain results in additional dimer-independent filamentation (Bigot *et al*., 2004). By comparison, in the *ftsK[blind]* strain, the frequency of filaments was only ∼3%, consistent with ∼70% of chromosomal dimers eventually being resolved. Comparable results for the extent of filamentation were obtained when equivalent cultures were examined microscopically (not shown).

A stringent test of strain fitness is provided by coculture competition assays (Bigot *et al*., 2004). Therefore, the fitness of *ftsK* variants when competing with the wild-type *ftsK* strain was tested over 40 generations. A reduction of fitness in this assay can result, in principle, from the production of inviable cells at division (for example, when unresolvable chromosome dimers occur) and/or by an increased generation time of cells in a population. The data indicate that most reduction in fitness arises from the production of inviable cells.

**Fig. 3:**
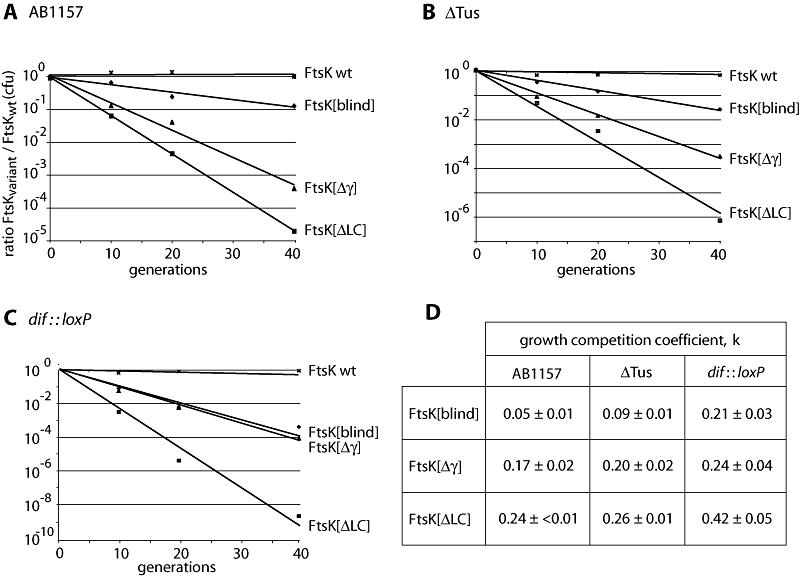
Growth competitions. The ratio of colony-forming units (cfu) obtained for a strain carrying the *ftsK* allele of interest to the cfu obtained for a strain with the wild-type *ftsK* allele in (A) an AB1157 background, (B) a Tus^−^ background, and (C) a *dif::loxP* background with cells expressing Cre recombinase. D. The growth competition coefficients obtained from assays shown in A–C (mean and SD of two experiments).

All variants demonstrated a growth disadvantage against the wild-type strain. Despite *ftsK[blind]* being the fittest variant strain, it had a reproducible growth disadvantage as compared with its wild-type competitor (Fig. 3A). If the growth disadvantage of *ftsK[blind]* were solely the result of production of inviable cells, then it would equate to a 5% probability of producing a non-viable cell/generation (*k*-value), whereas if it were due solely to a slower growth rate of viable individual cells it would equate to a mean cellular generation time difference of 10%. *ftsK[ΔLC]* was the least fit strain (*k* = 0.24), with *ftsK[Δγ]* having an intermediate fitness (*k* = 0.17), results consistent with the observed levels of filamentation (∼15%) and suggestive that the competitive disadvantage of both *ftsK[blind]* and *ftsK[Δγ]* arises from defects in chromosome dimer resolution. We infer that *ftsK[blind]* is ∼70% efficient in dimer resolution, whereas *ftsK[Δγ]* is completely defective. In support of this conclusion, the reduced fitness of *ftsK[blind]* and *ftsK[Δγ]* strains was suppressed in *xerD* and *recA* backgrounds (data not shown).

### The efficiency of XerCD–*dif* recombination is reduced in the *ftsK[blind]* strain

**Fig. 4:**
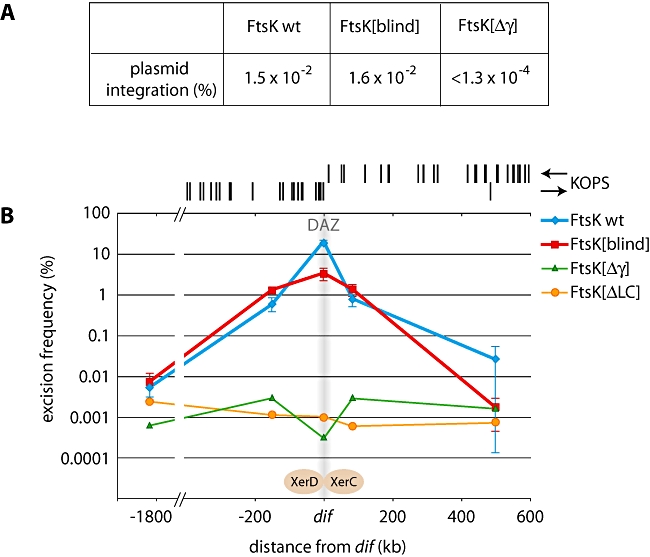
Efficiency of *dif* recombination. A. Plasmid integration assay. B. *dif-lacI-dif* cassette excision assay. A *dif-lacI-dif* cassette was inserted at five chromosomal loci in *ftsK*^+^ and *ftsK* variant strains. The frequency of cassette excision is plotted against the distance between the cassette position and the chromosomal *dif* locus. SD are indicated for FtsK wt and FtsK[blind]. The distribution of KOPS (GGGCAGGG) within the region −400 kb to +600 kb is shown above the plot. The orientation of *dif* is indicated by the marked XerD and XerC binding sites.

In order to confirm if the modest difference in growth rates and fitness of the *ftsK[blind]* strain indeed reflects a diminished ability to resolve chromosome dimers in a timely way, we analysed directly the capacity of this variant to support XerCD–*dif* recombination. First, we measured the rate at which a *dif*-containing plasmid, temperature-sensitive for replication, is integrated at the chromosomal *dif* locus in strains carrying the *ftsK* variants. The *ftsK[blind]* strain was found to be as efficient as an FtsK^+^ strain in promoting XerCD–*dif* recombination in this assay, whereas, as expected, the *ftsK[Δγ]* strain did not support plasmid integration (Fig. 4A).

We then monitored XerCD–*dif* recombination in a more discriminating assay that measures recombination at *dif* sites contained within a *dif-lacI-dif* cassette located at different positions in *ter* (Perals *et al*., 2000; C. Pages and F. Cornet, unpubl. data). In the FtsK^+^ strain, XerCD–mediated excision was 20% per generation when the cassette was inserted at the normal *dif* position, with the wild-type KOPS-loading sites directing FtsK towards *dif* on both sides (Fig. 4B). KOPS sequences are absent in the cassette. The excision frequency decreased 23-fold and 32-fold respectively, when the cassette was moved 85–150 kb away from the normal *dif* position. We assume this is because *dif* is now bounded by non-permissive KOPS binding sites on one side, and therefore outside of the ‘*dif* activity zone’, DAZ. These results are in agreement with earlier observations that characterized DAZ genetically (Perals *et al*., 2000). When the *dif* cassette was moved > 500 kb away from the normal position, XerCD recombination had fallen to the background levels exhibited by translocase-deficient variants of FtsK, presumably because FtsK can no longer access this region of the chromosome from the septum.

In the *ftsK[blind]* strain, the efficiency of cassette excision was similar at *dif* and the immediate bounding positions 85 and 150 kb away, as expected for a translocase that is not directed by KOPS. The 2.5-fold higher excision frequency at *dif* may be because FtsK more efficiently accesses this region even in the absence of KOPS recognition. The sixfold difference between FtsK^+^ and *ftsK[blind]* strains in recombination at *dif* presumably reflects the contribution of KOPS loading to the efficiency of FtsK translocation towards *dif*, and is in general agreement with the reduced efficiency of chromosome dimer resolution by FtsK[blind], measured in the previous assays.

### KOPS-guided translocation is more important for fitness in the absence of Tus

The ability of FtsK to translocate chromosomal DNA appears to be restricted to the period of septum closure (Kennedy *et al*., 2008). We reasoned that increasing the need for this interaction by interfering with timely chromosome segregation would therefore reveal a more pronounced phenotype in *ftsK[blind]* cells. In order to explore this possibility, we used, here and in the following sections, mutant strains expected to be compromised for chromosome segregation and/or organization.

In *E. coli*, Tus binding to *ter* sites helps ensure that replication termination occurs within the terminus region (reviewed in Neylon *et al*., 2005). The absence of Tus does not lead to an obvious strong phenotype (Morris *et al*., 1985). Nevertheless, a fraction of cells may over-replicate chromosomal DNA as a consequence of inappropriate terminations, and this may lead to increased recombination and chromosome dimer formation (Markovitz, 2005). Similarly, in *B*. *subtilis*, deletion of the replication termination protein, Rtp, has been implicated in an increase in chromosome dimer formation (Lemon *et al*., 2001). We therefore explored the possibility that the fitness of FtsK variants might be additionally compromised in the absence of Tus.

*FtsK[blind]*, *ftsK[Δγ]* and *ftsK[ΔLC]* variants lacking Tus were individually grown in competition against a FtsK^+^ Tus^−^ strain. The general trend of relative fitness between all strains was maintained (Fig. 3B). The *k*-value for all *ftsK* variant strains increased slightly, demonstrating that the requirement for the γ subdomain to direct DNA translocation by FtsK increases modestly in the absence of Tus. We conclude that Tus^−^ cells generate a slightly higher frequency of dimers in a population, and the probability of FtsK[blind] resolving such dimers is reduced to ∼55%; nevertheless a differential fitness between *ftsK* alleles remains (Fig. 3D). We believe this reduced efficiency arises when FtsK at the septum has to translocate much further to engage *dif* in cells in which replication termination occurs at a position distant from *ter*, thereby leading to its mispositioning.

### KOPS-guided DNA translocation is required to ensure formation of simple synapses during chromosome dimer resolution

Cre–*loxP* recombination can substitute for XerCD–*dif* recombination within *ter* to resolve chromosome dimers (Leslie and Sherratt, 1995; Capiaux *et al*., 2002), and is reflected by no growth disadvantage of *dif::loxP* cells in a coculture assay with *dif* cells (data not shown). Although Cre–*loxP* recombination does not require activation by FtsK, FtsK translocation simplifies the products of Cre–*loxP* recombination *in vitro* by facilitating simple synapsis between the recombining sites, thereby preventing the formation of complex recombinant products (Ip *et al*., 2003). Consistent with this, chromosome dimer resolution mediated *in vivo* by Cre–*loxP* depends on FtsK, presumably because its translocation prevents the formation of unsegregatable knotted and catenated chromosomes (Capiaux *et al*., 2002; Grainge *et al*., 2007). We therefore anticipated that KOPS-guided DNA translocation by FtsK might be crucial for chromosome unlinking in a Cre^+^*dif::loxP* strain. Because Cre–l*oxP* recombination is not dependent on activation by FtsK, we also predicted that *ftsK[Δγ]* and *ftsK[blind]* might be equally impaired in Cre–*loxP*-mediated chromosome dimer resolution, although it is possible that the lack of a γ subdomain in FtsK[Δγ] leads to it loading less well onto DNA than FtsK[blind].

Coculture experiments were set up between each *ftsK* variant and wild-type *ftsK* in the *dif::loxP* background, in which *dif* was substituted by *loxP* and Cre was expressed constitutively from a plasmid. Strikingly, *ftsK[Δγ]* and *ftsK[blind]* showed a similar growth disadvantage relative to the wild-type allele (Fig. 3C). This supports the view that the difference in fitness between these two alleles in cells competent for XerCD–*dif* recombination (Fig. 3A) is accounted for by the inability of FtsK[Δγ] to activate XerCD–*dif* recombination. Furthermore, the data suggest that the ability of Cre–*loxP* to substitute for XerCD–*dif* in dimer resolution is totally dependent on KOPS-guided DNA translocation by FtsK, because dimers are expected to form by homologous recombination at the same frequency in *dif* and *dif::loxP* strains. These data support the view that the efficiency of FtsK translocation needs to be optimal during chromosome dimer resolution by Cre–*loxP* in order to avoid FtsK-independent recombination events on complex synapses, which would generate topologically complex products. The observation that the *k*-values for all three *ftsK* variants in a *dif::loxP* strain were higher than for the *ftsK[Δγ]* AB1157 strain (Fig. 3D) suggests that chromosome unlinking in these strains has additional problems caused by Cre–*loxP* recombination itself. We believe this is likely to be the consequence of the production of topologically complex products, which are avoided in a FtsK^+^*dif::loxP* strain because KOPS-guided translocation effectively ensures that all recombination takes place within simple synapses. The smaller decrease in fitness between *ftsK[Δγ]* cells in the XerCD–*dif* and the Cre–*loxP* background (Δk of 0.07) compared with *ftsK[ΔLC]* cells in these backgrounds (Δk of 0.18) suggests that non-directed FtsK translocase activity remains advantageous in the Cre–*loxP* background, presumably because it is still able to limit chromosomal entanglement during Cre–*loxP* recombination.

Finally, as compared with the Tus^−^ situation, FtsK[blind] has a much reduced ability to unlink chromosome dimers and entangled chromosomes when Cre–*loxP* recombination is substituting for XerCD–*dif* recombination.

### DNA translocation by FtsK is crucial for survival of *muk* cells

*Escherichia coli* MukBEF acts in chromosome organization, and impairment of MukBEF leads to temperature sensitivity, disorganized chromosomes and the production of anucleate cells, apparently because of a failure to segregate newly replicated chromosomes efficiently (Niki *et al*., 1991; Britton *et al*., 1998; Danilova *et al*., 2007). *mukBEF ftsK[ΔC]* strains are synthetically lethal (Yu *et al*., 1998), as are *smc SpoIIIE* strains of *B. subtilis* (Britton and Grossman, 1999). Possible explanations of this synthetic lethality are: accumulative decreases in negative supercoiling, an increased requirement for chromosome translocation by FtsK/SpoIIIE to complete chromosome segregation in strains containing disorganized sister chromosomes, or an increased requirement for site-specific recombination to unlink replicated chromosomes; for example, because of increased chromosome dimer formation. We therefore investigated whether KOPS-guided DNA translocation by FtsK is central to the survival of a *mukBEF* strain.

**Fig. 5:**
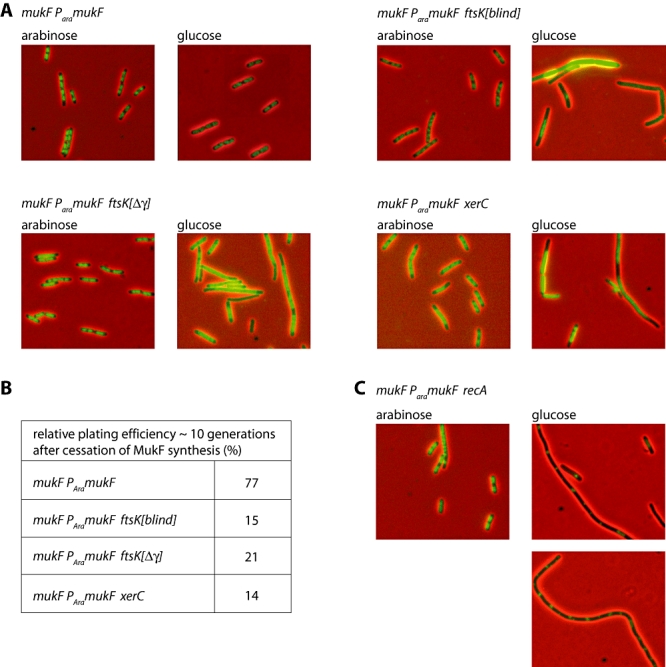
Phenotype of *ftsK* variants in *mukF P*_*ara*_*mukF* cells. A. Microscopy of *mukF P*_*ara*_*mukF ftsK* variants and *mukF P*_*ara*_*mukF xerC* grown at 22°C in LB supplemented with arabinose or glucose was performed after staining the cells with 4,6-diamidino-2-phenylindole (green) to visualize DNA. B. The relative plating efficiency of *mukF P*_*ara*_*mukF ftsK* variants and *mukF P*_*ara*_*mukF xerC* after ∼10 generations of exponential growth at 22°C in LB in the presence of either glucose or arabinose. C. Microscopy of *mukF P*_*ara*_*mukF recA* cells grown at 22°C in LB in the presence and absence of arabinose.

A strain mutated for its endogenous *mukF* gene, and carrying an inducible ectopic chromosomal copy of *mukF* under the control of the arabinose promoter (*mukFP*_*ara*_*mukF*) was characterized. The strain grew in rich medium at non-permissive temperature (37°C) only in the presence of arabinose, confirming an inducible Muk^+^ phenotype. In the absence of arabinose, *mukFP*_*ara*_*mukF* grew at the permissive temperature (22°C), but its viability was reduced. There was a 23% drop in colony-forming units (cfu) after 10 generations of growth at 22°C in LB lacking arabinose as compared with growth with arabinose (Fig. 5B). This is consistent with a proportion of divisions giving rise to anucleate cells at permissive temperatures in *muk* strains (Niki *et al*., 1991; Britton *et al*., 1998). We subsequently introduced *ftsK[blind]*, *ftsK[Δγ]* and *ftsK[ΔC]* alleles, along with an ATPase mutant allele (D1121N), referred hereafter as *ftsK[WalkerB]*, into the *mukFP*_*ara*_*mukF* strain. In the presence of arabinose, the *mukFP*_*ara*_*mukF* strains carrying the four different *ftsK* alleles had a Muk^+^ phenotype at 37°C.

In the absence of arabinose, *mukF P*_*ara*_*mukF ftsK[blind]* and *mukF P*_*ara*_*mukF ftsK[Δγ]* strains were viable at 22°C, although they produced small colonies. Assessment of cfu in cultures grown at permissive temperature for 10 generations without arabinose showed a 79–85% drop in viability as compared with cultures grown in arabinose, while microscopic analysis revealed extensive filamentation and unsegregated chromosomes (Fig. 5A and B). This phenotype is consistent with an increased demand for directed DNA translocation by FtsK when chromosome organization and segregation are disrupted. A similar drop in viability was observed in a *mukF P*_*ara*_*mukF xerC* strain, in which DNA translocation by FtsK is normal. Microscopy of this strain also revealed extensive filamentation and unsegregated chromosomes when grown at 22°C without arabinose (Fig. 5A and B). The similarities in phenotype of *xerC*, *ftsK[blind]* and *ftsK[Δγ]* strains in the absence of MukF indicate that loss of KOPS-guided DNA translocation by FtsK severely reduces the ability of FtsK to support chromosome dimer resolution, exacerbating the problems of chromosome segregation in Muk^−^ cells.

Consistent with a previous report (Yu *et al*., 1998), the *mukF P*_*ara*_*mukF ftsK[ΔC]* strain was inviable at 22°C in the absence of arabinose. Similarly, the *mukF P*_*ara*_*mukF ftsK[WalkerB]* was inviable in these conditions, demonstrating that it is the loss of DNA translocation activity rather than the loss of the C-terminal domain that results in the lethality of the double mutants. We conclude that ‘blind’ DNA translocation contributes to chromosome segregation in *muk* cells and that the absence of FtsK translocation leads to synthetic lethality in such cells.

### Absence of MukF leads to increased chromosome dimers

The extensive filamentation at 22°C of the *mukF P*_*ara*_*mukF xerC* strain in the absence of arabinose was suppressed when MukF was expressed (Fig. 5A). This suggests strongly that MukF^−^ cells form dimers more frequently. In order to test this directly, the frequency of excision of a *dif-Cm-dif* cassette positioned at the *dif* locus was assayed; excision frequency rises in concert with rising dimer formation. When grown in the absence of arabinose, the *mukF P*_*ara*_*mukF* strain excised the cassette at a frequency of 41% per generation, about twofold higher than the 20% excision rate in the control strain (Fig. 4B), consistent with chromosome dimers occurring more frequently in the absence of MukF. In the presence of arabinose, the excision rate in the *mukF P*_*Ara*_*mukF* strain was 31%, suggesting that suppression of the MukF^−^ phenotype by ectopic MukF expression is not complete.

Chromosome dimers form as a consequence of RecA-mediated homologous recombination. We anticipated that any increase in dimeric chromosome formation in Muk^−^ cells could reflect increases in DNA damage and recombinational repair in such cells (as inferred by Kouzminova *et al*., 2004), or simply an increase in interaction between homologous regions in sisters as a consequence of changes in chromosome organization. In the former case, introduction of a *recA* allele into Muk^−^ cells would further decrease viability because of an inability to repair potentially lethal DNA damage, while in the latter case the presence of a *recA* allele would suppress the defects arising from dimer formation in Muk^−^ cells.

In order to distinguish these alternatives, a *mukF P*_*ara*_*mukF* strain was made *recA* in the presence of arabinose. Upon removal of arabinose, the strain formed very small colonies at 22°C. 4,6-diamidino-2-phenylindole-stained cells showed extensive filamentation, with most filaments containing very little DNA stain, indicative of extensive DNA degradation (Fig. 5C). This finding supports the hypothesis that in the absence of functional MukBEF, cells accumulate DNA damage, whose repair demands RecA processing, with consequent increased chromosome dimer formation.

In conclusion, a lack of KOPS-guided FtsK translocation in *muk* cells compromises viability in a cell population for two reasons. First, these populations have an increased frequency of chromosome dimer formation; nevertheless, preventing dimer formation by making the *muk* cells *recA* does not suppress this phenotype because of increased need for recombinational repair in *muk* cells. Second, the probability of resolving dimers is much reduced, as evidenced by the similarity of the phenotype when either *ftsK[blind]* or *xerC* alleles are present in the absence of functional MukBEF. We believe it likely that this reduced efficiency of unlinking in individual cells arises because of the aberrant chromosome organization in *muk* cells (Danilova *et al*., 2007), thereby requiring more extensive FtsK translocation at the septum in order to access *dif*.

### Conclusions and perspective

We have shown that defects in KOPS recognition in otherwise healthy cells have little effect other than a decrease in the efficiency of chromosome dimer resolution and unlinking; we estimate that ∼70% of chromosome dimers are resolved to monomers in cells that are wild-type other than carrying the *ftsK[blind]* allele (Fig. 3D). KOPS guides FtsK translocation by acting as a directional loading site for FtsK. ‘Non-permissive’ KOPS do not stop or stall FtsK translocation; rather non-permissivity arises probably when an FtsK hexamer loaded onto a non-permissive site collides with the reporter FtsK molecule that is translocating towards it (Bigot *et al*., 2006; Löwe *et al*., 2008). A consequence of this is that interaction of FtsK with KOPS does not need to lead to downregulation or dissociation of the translocase. In the absence of KOPS, or KOPS recognition, FtsK can still load at random sites, from where it can translocate processively. In the absence of KOPS recognition, loading should be less efficient and 50% of FtsK loading events in *ter* should still be in a direction that favours resolution of chromosome dimers. If FtsK is present in excess and random loading onto DNA is sufficiently efficient, it may lead to frequent clashes between FtsK motors translocating in opposite directions. Nevertheless, loading by FtsK[blind] is clearly efficient enough, and clashes infrequent enough, to ensure efficient XerCD–*dif* recombination.

The data show that FtsK can be loaded, irrespectively of KOPS recognition, at least 85 kb from the XerC side of *dif* and 150 kb from the XerD side. In contrast, sites 500 kb from the XerC side of *dif* and 1800 kb to the XerD side appear unable to load FtsK. Consistent with this, other experiments show that the FtsK ‘capture zone’ is ∼400 kb (Corre and Louarn, 2005; C. Pages and F. Cornet, unpubl. data). The much smaller genetically defined ‘*dif* activity zone’, DAZ, which corresponds to ∼10 kb either side of *dif* locus (Perals *et al*., 2000), has been inferred to be the region within which directed FtsK translocation from either side can efficiently access chromosomal *dif*. Nevertheless, DAZ appears not to be precisely defined by KOPS, because within 10 kb of chromosomal *dif* there are two GGGCAGGG KOPS on the XerD side (124 bp, 7893 bp, away from *dif*) and none on the XerC side. If the KOPS consensus GGGNAGGG is used, this increases to three and two KOPS, respectively, within DAZ. We therefore infer that a single KOPS directing FtsK away from *dif* may be insufficient to ablate recombination at *dif*, and up to three KOPS directing FtsK away from *dif* are needed to efficiently prevent FtsK access to *dif in vivo*.

The failure of FtsK[blind] to recognize KOPS has more serious consequences for a cell population when chromosome dimers occur more frequently. Furthermore, the efficiency with which dimers are unlinked to monomers in individual cells is also less efficient in the presence of FtsK[blind] when perturbations to chromosome organization and processing were introduced by removing functional MukBEF; by deleting the replication terminator, Tus, which acts to direct replication termination to *ter*; or by substituting Cre–*loxP* recombination for XerCD–*dif* in dimer resolution. Loss of functional MukBEF and dimer resolution by Cre–*loxP* recombination had particularly strong consequences when FtsK[blind] replaced wild-type FtsK. These observations complement and add to our previous demonstration that KOPS-guided translocation by FtsK is essential if XerCD–*dif* recombination is to successfully decatenate newly replicated chromosomes in the absence of topoisomerase IV (Grainge *et al*., 2007).

We propose that the requirement for directed translocation by FtsK for efficient dimer resolution in Tus^−^ and MukBEF^−^ individual cells arises as a consequence of chromosome disorganization in these strains leading to sister *dif* sites being distant from septal FtsK during the late steps of the cell cycle; thereby requiring extensive directed translocation if chromosome unlinking is to occur; the data indicate that disorganization is greater in *mukBEF* cells. In contrast, when Cre–*loxP* recombination replaces that by XerCD–*dif*, chromosome organization appears normal, but local DNA translocation by FtsK ensures that sister *loxP* sites are brought together in a simple synapse, which does not entrap any intervening DNA, thereby ensuring that the site-specific recombination reaction gives unlinked products (Ip *et al*., 2003; Grainge *et al*., 2007). The mechanism by which entrapped DNA is removed between recombination sites during translocation remains unclear, although the process is evident *in vitro* in both Cre–*loxP* recombination and XerCD–*dif* recombination; in the latter case recombination is restricted to simple synapses.

Future challenges are to understand the molecular organization of FtsK at the septum, and to develop ‘almost real-time’ methods that allow visualization of individual FtsK molecule loading, translocation and activation of chromosome unlinking in live cells.

## Experimental procedures

### Bacterial strains

*ftsK[ΔLC]* lacks the linker and the C-terminal translocase domain (deleted for aa residues 211–1329; Bigot *et al*., 2004); The *ftsK[Δγ]* lacks residues 1248–1329. *ftsK::cat1*, which carries a Tn10-*cat* insertion in the linker domain and expresses the essential N-terminal domain of *ftsK* (Diez *et al*., 1997), was used in *in vivo* assays to test function of FtsK_50C_ variants (in DS9041) and for purification of FtsK_50C_ and FtsK_50C_ variants and in strains constructed for studying *muk* phenotype; it provides the *ftsK(ΔC)* allele. Strains for the *dif-lacI-dif* excision assay were derived from LN2666 (W1485 F^−^*leu thyA thi deo supE rpsL*; Cornet *et al.*, 1994). Strains for studying the *muk* phenotype were derived from AB1157 *mukF::kan* (Yamanaka *et al*., 1996), and contained *P*_*ara*_*mukF-frt::ΔargE.* AB1157 was used as the parental strain in all other experiments. Alleles were transferred by P1 transduction.

### Proteins

N-terminal Flag-epitope tagged EcFtsK_50C_, FtsK_50C_αβ and FtsK_50C_γ variants were expressed from the arabinose promoter of plasmid pBAD24 and purified on M2 anti-flag agarose affinity gel (Sigma). XerC and XerD proteins were purified as in Ferreira *et al*. (2001).

### *In vivo* and *in vitro dif* plasmid recombination and triplex displacement

The reactions were performed as in Sivanathan *et al*. (2006). The KOPS-2 plasmid had two directly repeated *dif* sites, each bounded by three overlapping KOPS in the non-permissive orientation (Sivanathan *et al*., 2006).

### Flow cytometry

Approximately 4 × 10^7^ exponentially growing cells were fixed in 70% EtOH and resuspended in PBS supplemented with Cyto16 stain. Samples were analysed on a FACSCalibur flow cytometer. Cyto16 fluorescence was detected by FL-1 and a lower cut-off value was set to exclude debris not containing DNA.

### Growth competition

A 1:1 mixture of two strains was grown in serial culture in LB at 37°C over 40 generations, and the relative frequencies of the two strains were determined by plating every 10 generations. The fitness coefficient or percentage of abortive divisions were calculated as described in Perals *et al*. (2000). In the *dif::loxP* background, Cre was expressed constitutively from pFX71 (pBAD18-Cre; Capiaux *et al*., 2002).

### Plasmid integration

Plasmid integration assays were performed by transforming the strain to be tested with pOL09, a thermosensitive vector carrying a *dif* site, and selected for at 30°C. Five colonies from this transformation were then inoculated into a single tube of LB medium and grown for 16 h at 30°C. Dilutions were then plated at 30°C and 42°C. pOL09 can persist at the elevated temperature only if it is integrated into the chromosome by XerCD-mediated recombination at chromosomal *dif*. The rate of integration was calculated from the ratio of cfu at 42°C to that at 30°C.

### *dif-lacI-dif* and *dif-Cm-dif* cassette excision

Strains carried a *xerC::Gm* mutation and the *dif-lacI-dif* or *dif*-Cm-*dif* cassette were made XerC^+^ by transformation with the appropriate Sp^R^ plasmid, pFC241. Transformants were plated on LB containing 20 μg ml^−1^ spectinomycin and grown overnight at 37°C. Five independent transformants were resuspended in LB plus spectinomycin, grown 5 h, diluted and plated on LB plus Xgal. The whole procedure corresponds to 20 generations before plating on LB–Xgal plates. The ratio of dark blue colonies on total was used to calculate the frequency of *lacI* loss per cell per generation. The average of five independent measures is shown. The *dif-Cm-dif* cassette excision in *mukF P*_*ara*_*mukF* strain assay was performed in a similar way apart from cultures being grown in LB supplemented with Cm and then diluted into LB supplemented with either arabinose or glucose and grown for 20 generations at 22°C, plated on LB plus arabinose, and monitored for the loss of Cm^R^.
